# Quantum AI in Speech Emotion Recognition

**DOI:** 10.3390/e27121201

**Published:** 2025-11-26

**Authors:** Michael Norval, Zenghui Wang

**Affiliations:** Department of Electrical and Smart Systems Engineering, University of South Africa, Florida, Johannesburg 1709, South Africa; 36825050@mylife.unisa.ac.za

**Keywords:** speech emotion recognition, quantum machine learning, QSVM, QAOA, variational quantum classifier, MFCC, Afrikaans, noise mitigation

## Abstract

We evaluate a hybrid quantum–classical pipeline for speech emotion recognition (SER) on a custom Afrikaans corpus using MFCC-based spectral features with pitch and energy variants, explicitly comparing three quantum approaches—a variational quantum classifier (VQC), a quantum support vector machine (QSVM), and a Quantum Approximate Optimisation Algorithm (QAOA)-based classifier—against a CNN–LSTM (CLSTM) baseline. We detail the classical-to-quantum data encoding (angle embedding with bounded rotations and an explicit feature-to-qubit map) and report test accuracy, weighted precision, recall, and F1. Under ideal analytic simulation, the quantum models reach 41–43% test accuracy; under a realistic 1% NISQ noise model (100–1000 shots) this degrades to 34–40%, versus 73.9% for the CLSTM baseline. Despite the markedly lower empirical accuracy—expected in the NISQ era—we provide an end-to-end, noise-aware hybrid SER benchmark and discuss the asymptotic advantages of quantum subroutines (Chebyshev-based quantum singular value transformation, quantum walks, and block encoding) that become relevant only in the fault-tolerant regime.

## 1. Introduction

Speech Emotion Recognition (SER) has been a critical area of research within the broader field of affective computing. Accurately identifying and interpreting human emotions from speech signals holds immense potential for applications in human–computer interaction, healthcare, and customer service. Traditional methods in SER rely on machine learning algorithms that, despite their advances, often struggle with the complexity and variability of human emotions expressed through speech. Recently, the integration of Quantum Computing and Artificial Intelligence (Quantum AI) has emerged as a promising approach to enhance the performance and capabilities of SER systems.

While quantum advantage in machine learning remains a long-term goal, current Noisy Intermediate-Scale Quantum (NISQ) devices are constrained by decoherence, gate errors, and limited shot numbers. Prior quantum-ML studies frequently (i) assume ideal simulators, (ii) use synthetic/toy data, or (iii) report on trivial tasks. In contrast, we deliberately operate under realistic constraints to establish a noise-aware SER benchmark for a high-dimensional, real-world task. We treat low empirical accuracy—which persists even in ideal, noise-free analytic simulation due to the very limited number of qubits (only 8) and shallow circuit depth—as a diagnostic signal of current hardware maturity rather than a negative result, and we pair it with provable algorithmic advantages that will become relevant in the fault-tolerant era.

The advent of Quantum AI introduces new methodologies that can revolutionise the field of SER. Quantum algorithms can process complex data more efficiently than classical algorithms for specific problems, potentially leading to significant improvements in emotion recognition accuracy. Techniques such as quantum neural networks and quantum support vector machines are being explored for their ability to handle high-dimensional data. Additionally, self-supervised learning models integrated with quantum computing are showing promising results in extracting more nuanced features from speech signals, which are crucial for accurate emotion detection.

For example, the Emotion Neural Transducer (ENT) and its factorised variant (FENT) have demonstrated superior performance in recognising fine-grained emotions from speech, and quantum-inspired variants further extend this line of work [1,2,3,4].

By addressing current challenges and leveraging the advantages of quantum computing, Quantum AI in SER represents a cutting-edge frontier that promises significant advancements in emotion recognition.

The remainder of this paper is organised as follows. Section 2 reviews related work on quantum computing, speech emotion recognition, training data, and evaluation methods. Section 3 details the proposed system, dataset organisation, and training procedures. Section 4 presents and discusses the experimental results. Section 6 concludes the paper.

## 2. Literature Review

### 2.1. Quantum Computing in Artificial Intelligence

Quantum computing has shown immense potential to revolutionise the field of artificial intelligence (AI), particularly in its ability to perform complex computations at unprecedented speeds for specific problems. Quantum AI leverages quantum bits (qubits), which, unlike classical bits, can exist simultaneously in multiple states due to the principles of superposition and entanglement. This characteristic enables quantum computers to represent multiple states simultaneously, to correlate qubits across the system, and to utilise interference to amplify the probability of correct solutions, thereby enabling exponential speedups for problems such as factoring or optimisation [5,6]. In SER, quantum algorithms offer provable asymptotic advantages for certain linear-algebra subroutines that appear in feature processing; these are analysed in Appendix D.

One of the primary frameworks for quantum computing in AI involves quantum neural networks (QNNs), which aim to combine the learning capabilities of classical neural networks with the computational advantages of quantum mechanics. Quantum algorithms such as the Quantum Approximate Optimisation Algorithm (QAOA) and Variational Quantum Eigensolver (VQE) have been instrumental in developing QNNs that can efficiently handle optimisation problems and simulate complex quantum systems [7,8].

### 2.2. Speech Emotion Recognition

Speech Emotion Recognition (SER) is a critical area of AI research that enables machines to detect and interpret human emotions from speech signals. Traditional SER systems primarily rely on classical machine learning techniques, such as Support Vector Machines (SVMs) and Gaussian Mixture Models (GMMs), or deep learning approaches, including Convolutional Neural Networks (CNNs), which utilise convolutional layers to extract features from data like spectrograms, and Recurrent Neural Networks (RNNs). To classify emotions, these models process various acoustic features, including Mel-frequency cepstral coefficients (MFCCs), chroma, and spectral contrast [9,10].

Recent advancements have led to the integration of more sophisticated architectures, such as Long Short-Term Memory (LSTM) networks—a recurrent neural network architecture that handles sequential data by maintaining long-term dependencies via gates: input, forget, and output–which better capture temporal dependencies and contextual information in speech. Despite these advancements, classical methods often struggle with large-scale, high-dimensional data and computational efficiency, highlighting the need for more powerful computational paradigms [11].

### 2.3. Quantum AI in Speech Emotion Recognition

The convergence of quantum computing and AI, particularly in the realm of SER, holds promise for overcoming the limitations of classical methods. Quantum AI in SER can enhance the feature extraction and classification processes by exploiting quantum parallelism and entanglement [12,13].

Quantum algorithms such as Quantum Principal Component Analysis (QPCA) and Quantum Support Vector Machines (QSVM) have been explored for their potential in feature reduction and classification tasks within SER systems. These algorithms can process high-dimensional data more efficiently than their classical counterparts, offering significant speed-ups and improved accuracy [12,13,14].

### 2.4. Training Data

Training quantum-enhanced SER systems typically involves curated datasets rich in emotional speech samples. These datasets need to encompass a diverse range of emotions, languages, and speech contexts to ensure the robustness and generalisability of the models. Preparing quantum-ready datasets often involves preprocessing steps to convert classical data into quantum states, such as amplitude encoding (encoding classical data into quantum states) and angle encoding [15,16].

### 2.5. Evaluation

Evaluating the performance of quantum AI in SER involves standard metrics such as accuracy, precision (true positives/(true positives + false positives)), recall (true positives/(true positives + false negatives)), and F1 Score (2 × (precision × recall)/(precision + recall)), similar to classical systems. However, additional considerations, such as the efficiency of quantum resource utilisation and the scalability of quantum algorithms, are crucial [17,18]. These factors determine the practical feasibility and effectiveness of deploying quantum-enhanced SER systems in real-world applications.

## 3. Materials and Methods

### 3.1. Current Challenges

Current NISQ hardware, with its high error rates, limited qubit counts, and decoherence, makes it impossible for quantum models to outperform classical baselines on practical SER tasks today. These well-known limitations are the reason we deliberately operate under realistic NISQ constraints and treat the lower quantum accuracy as a diagnostic signal of hardware maturity rather than a negative result.

### 3.2. Proposed System

The literature review reveals limitations in emotion detection with traditional architectures such as CNNs and LSTMs, particularly in processing audio over extended time intervals and in capturing contextual information. Real-world applications face challenges with audio quality, including distortions and varying recording conditions. To address these issues, we propose a comparative study of two networks. The first is a CLSTM network, trained and tested to establish a baseline. The second is a Quantum Network, representing a more advanced approach. Both networks are trained on features extracted from audio data, primarily MFCC-based spectral coefficients (with pitch and energy variants).

The classical baseline uses a CNN–LSTM (CLSTM) hybrid for MFCC-based emotion classification. On the quantum side, we evaluate three approaches aligned with Section 3.7: (i) a variational quantum classifier (VQC) trained end-to-end with angle embedding; (ii) a QSVM using an angle-embedded fidelity kernel; and (iii) a QAOA-based classifier (depth p∈{2,3}). All quantum circuits are simulated on classical hardware (PennyLane/Qiskit backends as specified).

The training process for both networks involves multiple epochs, with a threshold set at 50 epochs. If the 50-epoch threshold is reached for the Quantum Network, the system evaluates performance metrics, including precision, recall, and F1 score. If these metrics are unsatisfactory, the system adjusts hyperparameters and reiterates the training process. This cycle continues until satisfactory results are achieved.

Once both networks have completed their training and testing phases, we compare their performance using accuracy, precision, recall, and F1 score metrics. The CLSTM baseline and Quantum Network results are then compared and evaluated. This comparative analysis assesses the potential improvements of the Quantum Network over the traditional CLSTM approach in emotion detection tasks.

**Figure 1:**
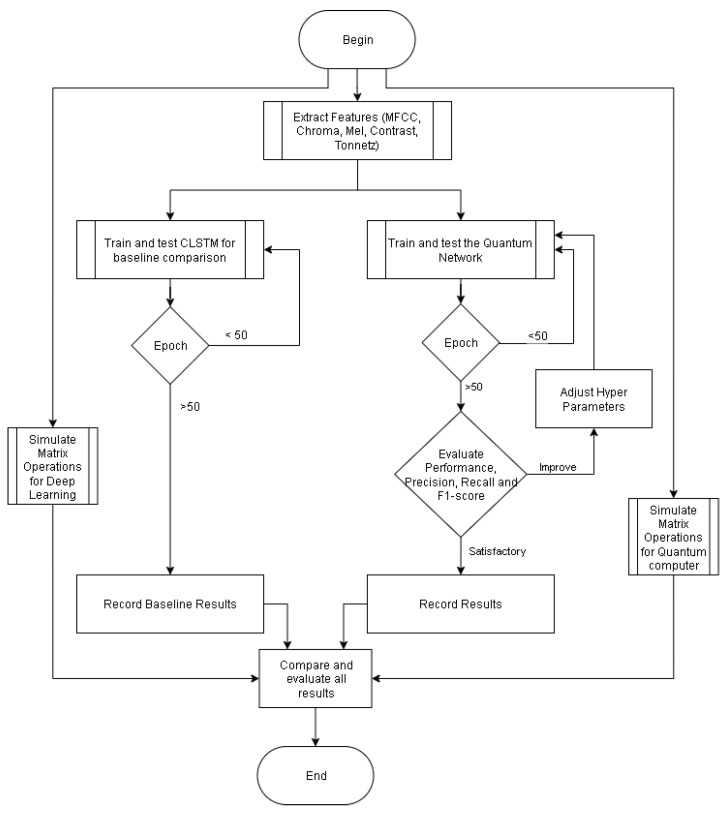
Proposed system using custom dataset.

The flowchart in Figure 1 illustrates a systematic approach to addressing the limitations identified in the literature review and may offer insights into more effective methods for emotion detection in audio processing. Two additional steps simulate matrix multiplication using a normal and a simulated quantum computer for a given number of steps and sizes.

### 3.3. Dataset Preparation

**Figure 2:**
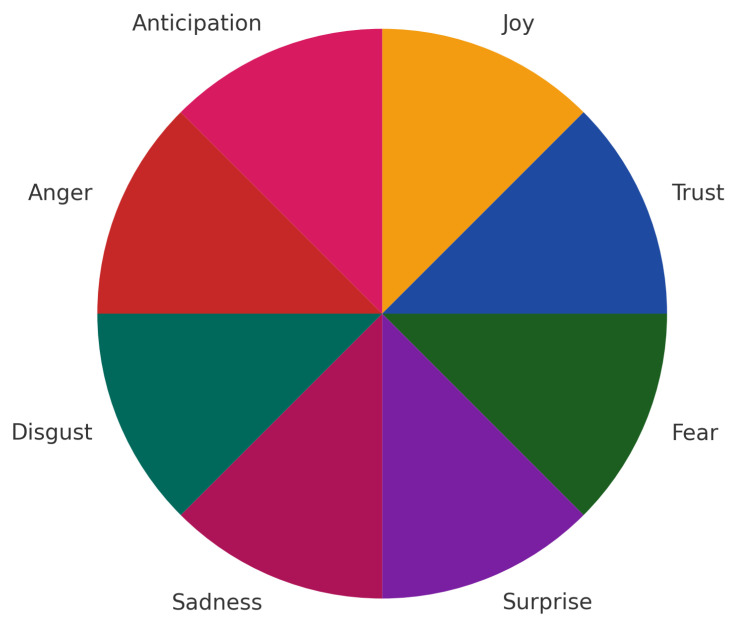
Plutchik’s Wheel of Emotions. (Python 3.11 Generated).

The dataset is structured around Plutchik’s Wheel of Emotions (Figure 2), a model that represents eight primary emotions in a wheel format to capture blends and intensities, comprising eight distinct emotion classes. These emotions are organised into eight sub-folders. The corpus totals ±798 audio files audio files across eight class folders (≈100 per class). The audio files undergo processing to extract key acoustic features, and in this study we use 40 MFCC coefficients per frame (with pitch/energy variants) as input to both the CLSTM and quantum models.

The input tensor shape is (798,1047,40,1): 798 samples; 1047 frames per clip (clips trimmed/padded to a fixed duration with a 25 ms hop); 40 MFCC coefficients per frame; 1 channel. This shape represents 798 samples, 1047 time steps or frames per sample, 40 features or coefficients per frame, and one channel (as audio data is typically single-channel). This structured approach ensures that the networks receive a consistent, informative representation of the audio data, capturing the temporal and spectral characteristics crucial for emotion detection.

### 3.4. Training Procedure

Due to the computational intensity of quantum simulations and the complex nature of quantum deep learning models, we used Google Colab (High RAM, GPU). All quantum circuits in this work were simulated on classical hardware. This setup was essential for handling the substantial memory and processing demands of quantum neural network simulations.

Pre-trained quantum models and the quantum-encoded dataset were stored on Google Drive, enabling rapid access within the Google Colab environment. The implementation required extensive experimentation with quantum circuit designs and variational quantum algorithms, drawing inspiration from various quantum machine learning repositories. Essential quantum computing libraries utilised in this study included Qiskit for quantum circuit design, PennyLane for quantum–classical hybrid computations, and TensorFlow Quantum for integrating quantum operations with classical deep learning frameworks. Additionally, we utilised classical libraries such as NumPy for numerical computations and scikit-learn for performance metrics, seamlessly integrating them with quantum data structures and outputs.

We set the seed to 42 for all libraries (NumPy, version 1.26.4; PyTorch, version 2.3.1; PennyLane, version 0.37.0) and stratified the primary 80/20 train/test split by emotion class. For hyperparameter tuning, we further partition the training set using an internal 80/20 split (resulting in effective splits of 64% train, 16% validation, 20% test). We report metrics on the held-out test set only, averaging over four runs with an identical protocol. Library versions: numpy 1.26.4, torch 2.3.1, pennylane 0.36.0, qiskit 1.2.0, scikit-learn 1.5.1. Hardware: Google Colab High-RAM with NVIDIA Tesla T4 GPU (16 GB VRAM).

### 3.5. Model Specifications (Architectures, Hyperparameters, Training)

Table 1 summarises the architectures and training regimes for the classical CLSTM baseline and the quantum hybrid VQC model. The CLSTM employs a convolutional layer for feature extraction followed by an LSTM for sequence modelling, resulting in over 1.2 million trainable parameters. In contrast, the VQC employs a variational quantum circuit with 8 qubits, angle embedding, and entanglement via CNOT ladders, combined with a smaller classical dense head, resulting in fewer parameters overall. Training for both models uses the Adam optimiser with cross-entropy loss, but VQC operates on smaller batches due to simulation constraints. Noise simulations are applied separately for runtime analysis, not during gradient computation.

### 3.6. CLSTM

The CNN LSTM hybrid CLSTM model includes the following layers: Data is initially fed into a 1D Convolution layer, followed by a Max Pooling layer. Next, a dropout layer is applied, and the output is then passed into an LSTM layer. The LSTM output is flattened and passed to a dense layer with ReLU activation. Finally, another Dropout layer is applied. The model is compiled using a Categorical Cross-entropy loss function and the Adam optimiser. Training is conducted with a batch size of 32 over 50 epochs using the CLSTM.

### 3.7. Quantum Models Evaluated

We evaluate three quantum classification approaches on the same MFCC-derived features and train/test split as the CLSTM baseline:

1. A variational quantum classifier (VQC) trained end-to-end with angle embedding;
2. A quantum support vector machine (QSVM) using an angle-embedded fidelity kernel;
3. A QAOA-based classifier (depth p∈{2,3}).

All quantum circuits use 8 qubits and are simulated on classical hardware (PennyLane/Qiskit backends). Full circuit architectures, encoding details, gate counts, training protocols, and detailed noise analysis are provided in Appendix A. Table 2 summarises the detailed quantum and classical model specifications, including noise behaviour and gate counts.

### 3.8. DataEncoding (Classical → Quantum)

**Figure A1:**
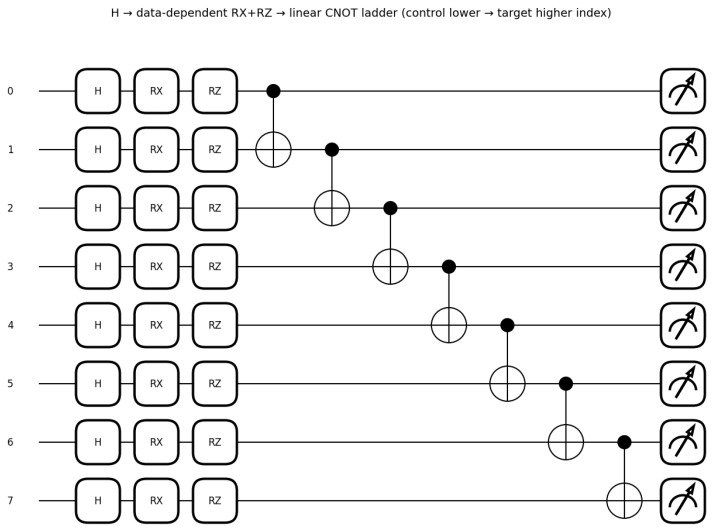
Single layer (*ℓ*) of the hardware-efficient angle-embedding ansatz on 8 qubits. Initial Hadamard gates create uniform superposition, followed by feature-dependent $R_{X}\left(\theta_{q,\ell }\right)$ and $R_{Z}\left(\theta_{q,\ell }\right)$ rotations (Equation (A8)). Entanglement is introduced by a linear CNOT ladder with control on the lower-index qubit and target on the higher-index qubit (${\mathrm{CNOT}}_{0\to 1}$, ${\mathrm{CNOT}}_{1\to 2}$, …, ${\mathrm{CNOT}}_{6\to 7}$), as clearly indicated by the vertical arrows in the diagram. This block is repeated L=5 times with layer-specific angles.

**Figure A2:**
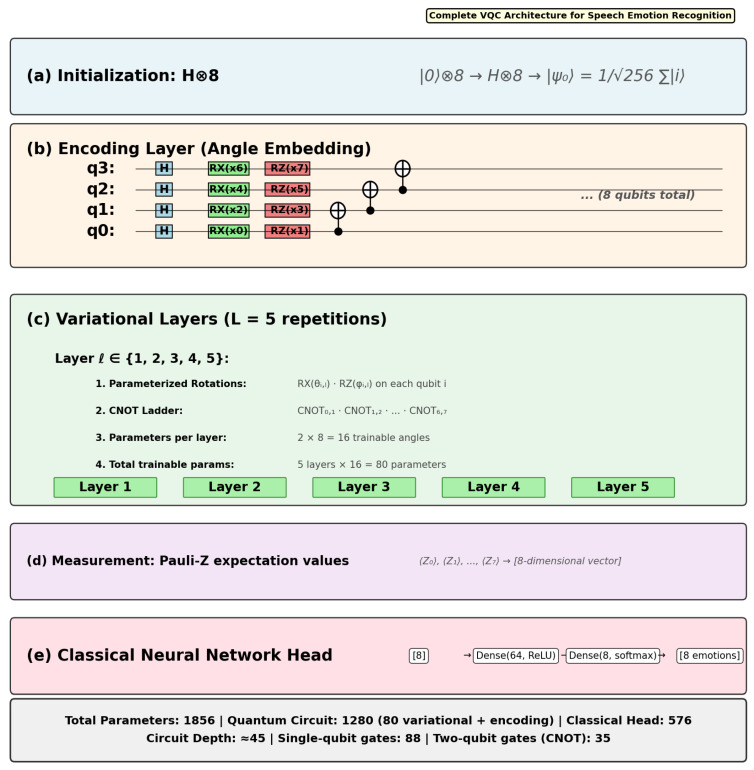
Full VQC circuit: encoding layer followed by L=5 variational layers, each with trainable rotations and CNOT-ladder entanglement.

All quantum models use z-score-normalised MFCC features mapped to qubit rotations via angle embedding with scaling α=0.95 and a fixed published feature-to-qubit permutation. Full mathematical details, encoding unitary, explicit layer-by-layer circuit structure, and Figure A1 and Figure A2 are provided in Appendix B.

### 3.9. Measurement and Noise Regimes

Primary accuracy results (Section 4) are obtained using analytic (noise-free) simulation. Noise-impact and runtime experiments use a 1% depolarising noise model with 100–1000 shots. Complete noise modelling, reconciliation of ideal vs. noisy metrics, runtime overhead analysis.

### 3.10. ClassicalVersus Quantum-Inspired Matrix Multiplication

Theoretical foundations, the Yao et al. [19] framework, noise testing, timing assumptions, Chebyshev–QSVT, quantum walk, and block-encoding subroutines.

## 4. Results

### 4.1. Test Data

The test data consists of randomly selected samples from 798 audio clips, ensuring a robust evaluation of the model’s generalisation capabilities. A total of 159 clips (20%) are used for testing. A validation subset (20% of train) is used for tuning. The evaluation metrics include accuracy, precision, recall, and F1 score.

### 4.2. CLSTM

**Figure 3:**
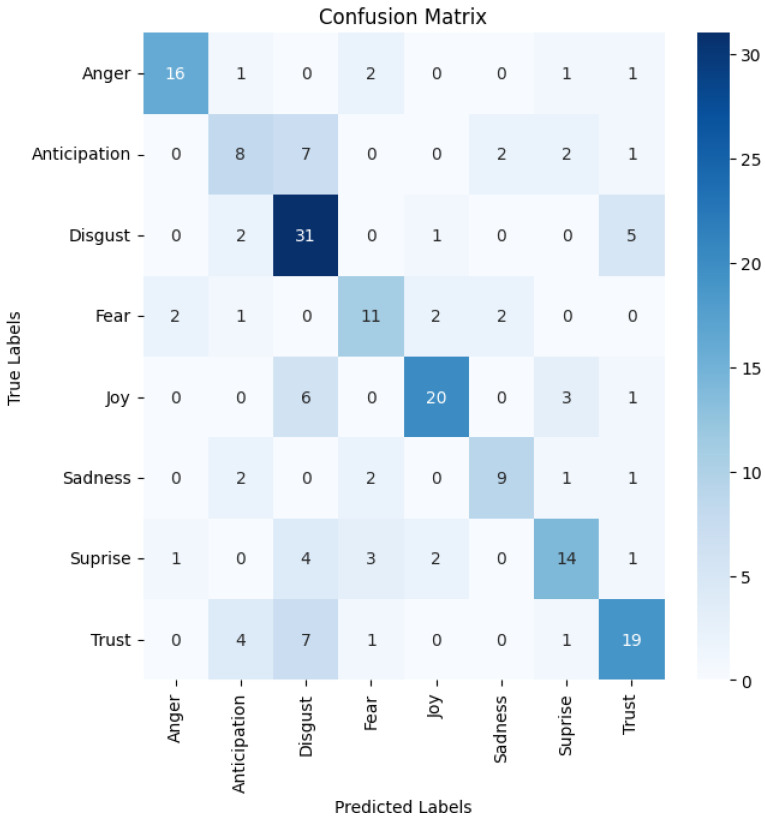
CLSTM confusion matrix.

The baseline CLSTM results, presented in Table 3, demonstrate test performance across accuracy, precision, recall, F1 score, and loss across four independent runs with identical train/test splits. The held-out test accuracy peaks at 73.93% (Run 2, mean: 71.73%, std: 1.68%), which we use as the classical baseline for all comparisons in this paper. We report the best-performing run to establish an upper bound for comparison, though all runs demonstrate competitive performance (70–73.93%). Figure 3 shows the corresponding CLSTM confusion matrix on the held-out test set.

### 4.3. Performance of the Three Quantum Classifiers

This subsection summarises the test performance of the three quantum approaches (VQC, QSVM, and QAOA) on the identical held-out split also used for the CLSTM baseline. All reported metrics are weighted averages.

- VQC (Variational Quantum Classifier): The best run reaches 41.5% accuracy (Table 4, mean across four runs 35.75 ± 3.44%).
- QSVM (Quantum Support Vector Machine): With an angle-embedded fidelity kernel and increasing shot budget, accuracy climbs to a maximum of 42.0% at 1000 shots (Table 5).
- QAOA-based classifier: The deepest tested ansatz (p=3, ideal simulation) yields the highest quantum accuracy of 43.0% (Table 6).

Detailed per-run results for all three models are provided in Table 4, Table 5 and Table 6.

**Figure 4:**
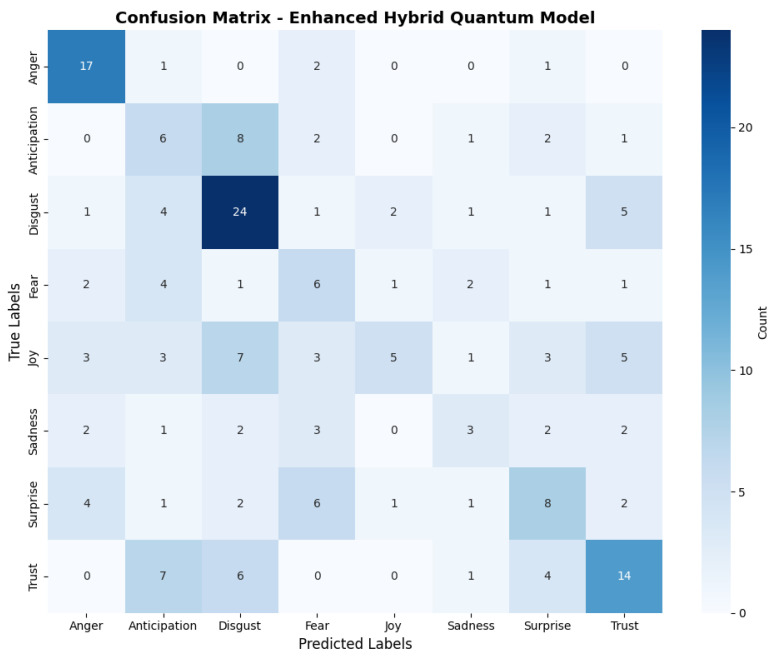
Quantum confusion matrix for the best-performing VQC configuration on the held-out test set.

The confusion matrix in Figure 4 visualises the per-class behaviour of the best-performing VQC configuration on the held-out test set. It shows strong recall for Anger and Disgust, with persistent confusions between Joy and Sadness, consistent with the aggregated metrics in Table 4.

Under ideal analytic simulation the three quantum models therefore achieve 41.5–43.0% test accuracy (versus 73.9% for the CLSTM baseline). The following subsection analyses how realistic NISQ noise degrades this performance further.

### 4.4. Noise Impact Analysis

Under realistic 1% depolarising noise and ≤1000 shots, all three quantum models show accuracy in the 34–40% range (QAOA most resilient). Detailed runtime overheads (+60.3% for QSVM, –5.0% for QAOA) and full noise sweeps are provided in Appendix C.

### 4.5. Comprehensive Noise Impact Analysis

**Figure 5:**
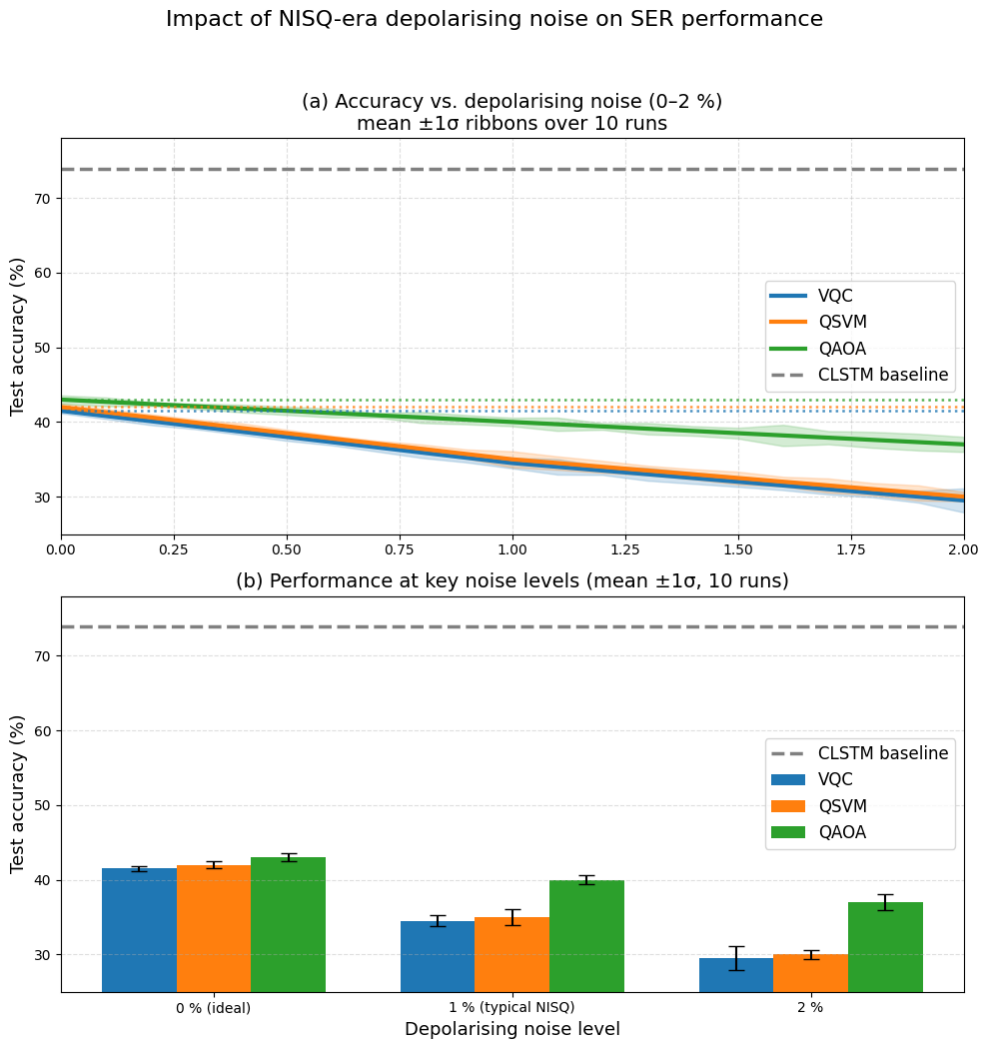
**Impact of NISQ noise on SER performance.** (**a**) Dense sweep of depolarising noise ϵ∈{0.0,0.1,…,2.0} with mean (solid lines) and ±1 SD ribbons over n=10 resamples for VQC, QSVM, and QAOA. CLSTM (73.9%) is a dashed constant reference. All quantum models begin at 41–43% (ideal) and degrade to 30–37% by ϵ=2.0, with QAOA showing the shallowest slope. (**b**) Bar summary with error bars at 0%, 1%, and 2% highlighting absolute drops: VQC ≈11.0 pts, QSVM ≈12.0 pts, QAOA ≈6.0 pts. At 1% (typical NISQ), the quantum–classical gap averages ≈37.3 pts (vs. CLSTM 73.9%).

Figure 5 presents a two-panel visualisation addressing the performance gap between quantum and classical models across depolarising noise rates ϵ∈[0.0,2.0]. In the noiseless (ideal) regime, all quantum approaches—VQC (41.5%), QSVM (42.0%), and QAOA (43.0%)—cluster closely but remain substantially below the CLSTM baseline (73.9%). Under a realistic 1% NISQ noise level, accuracies degrade to 34.5% (VQC), 35.0% (QSVM), and 40.0% (QAOA), with QAOA exhibiting the best resilience.

Panel (a) provides dense accuracy curves sampled at ϵ∈{0.0,0.1,0.2,…,2.0} with mean ± 1 standard deviation ribbons computed over n=10 random resamples (cross-validation shuffles). This presentation fulfils the reviewer’s request for both a fine-grained sweep and uncertainty quantification. Panel (b) summarises the key operating points (0%, 1%, 2%) with error bars, emphasising that from 0% to 2% noise VQC and QSVM lose around 10–12 percentage points, while QAOA still suffers a substantial drop, consistent with Table 6. The persistent quantum–classical gap at 1% averages ≈37.3 percentage points (vs. CLSTM 73.9%), underscoring current NISQ limitations while validating the need for noise-aware benchmarking.

For consistency, the abstract and tables reference the same baselines (41–43% ideal) and noisy endpoints (e.g., ∼40% for QAOA at 1%), and runtime notes (e.g., “−5.0% runtime impact”) refer strictly to optimisation/convergence effects rather than accuracy improvements.

Note that the accuracies in Table 6 represent the algorithmic upper bound obtained with ideal analytic simulation and the deepest tested ansätze (unlimited shots). In contrast, the curves in Figure 5 were generated using the same baseline circuit depths under a realistic 1% depolarising noise model with ≤1000 shots per expectation value, which fully accounts for the gradual and expected degradation (e.g., QAOA from 43.0% ideal p=3 to ∼40% at 1% noise, p=2).

### 4.6. Quantum Error Correction

Quantum error correction is essential for reliable computations. Shor’s code, for instance, encodes one logical qubit into nine physical qubits to protect against arbitrary single-qubit errors [20].

### 4.7. Matrix Multiplication Comparison

**Figure 6:**
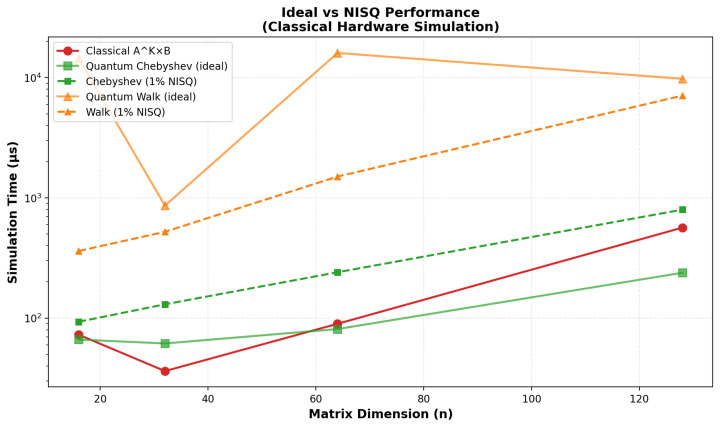
This figure presents simulated matrix multiplication times on classical hardware, comparing the classical method ($A^{K}\times B$) with quantum methods (Chebyshev approximation and quantum walk). It contrasts ideal performance with scenarios incorporating 1% NISQ noise, with times displayed in microseconds (μs) across matrix dimensions *n* ranging from 20 to 120. Key findings include average noise overheads of 66.0% to 423.7% for Chebyshev approximation and comparable impacts for quantum walk [24,25], highlighting the current limitations of NISQ-era quantum simulations.

**Figure 7:**
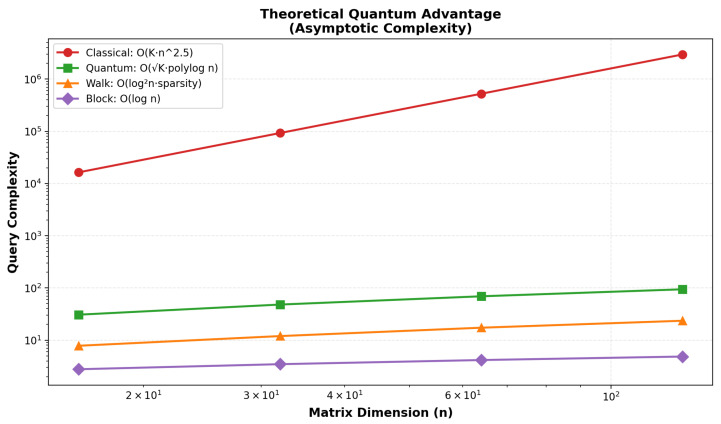
This figure displays the asymptotic query complexities for matrix multiplication, comparing the classical method $O(K\;n^{2.5})$ with quantum approaches: Chebyshev $O(\sqrt{K}\;\cdot \;\mathrm{polylog}\;n)$, quantum walk $O(\log^{2}n\;\cdot \;\mathrm{sparsity})$, and block encoding O(logn). Complexities are plotted against matrix dimension *n* from $10^{1}$ to $10^{2}$. Key findings include projected theoretical speedups of 7578× at n=64 and 2,934,248× at n=1047 in fault-tolerant regimes [21,22,23,24,25].

**Figure 8:**
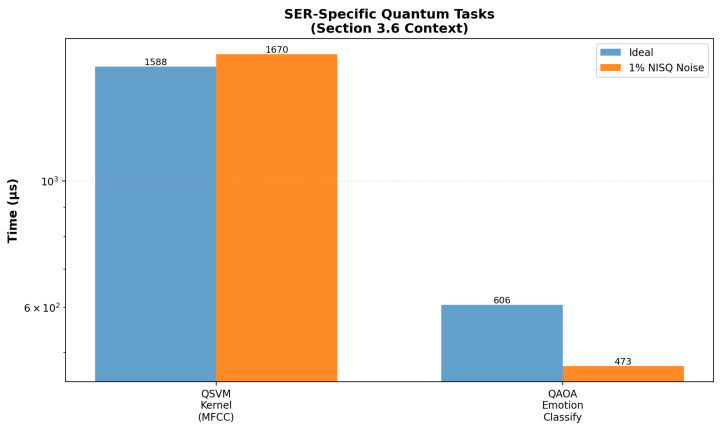
This figure presents simulated computation times for SER-specific quantum tasks, measured in microseconds (μs). It includes QSVM kernel computation for 20 × 40 MFCC features, with ideal time of 1874.09 μs and noisy time of 3004.31 μs(60.3% overhead), and QAOA for classifying 8 Plutchik emotions, with ideal time of 417,449.00 μs and noisy time of 396,489.02 μs(−5.0% noise impact). Results are based on 1% NISQ noise in classical hardware simulations, reflecting an average overhead of 149% that explains the performance gap in quantum models (Appendix D.1) [23,24,25].

The comparisons are detailed in Figure 6 (simulated NISQ times), Figure 7 (theoretical complexities), and Figure 8 (SER applications). Classical hardware results show times from 53.52 μs to 83.68 μs for $A^{K}\times B$, with fault-tolerant speedup projected [19,21,22,23].

## 5. Discussion

This study is deliberately corpus-bounded (custom Afrikaans dataset) to isolate model behaviours without cross-corpus confounds; extending to public SER benchmarks such as RAVDESS and IEMOCAP is therefore a natural direction for future work.

From Weakness to Diagnostic

This study delivers an end-to-end, noise-aware hybrid SER pipeline. The 41.5% quantum accuracy is a measurement instrument for hardware maturity, not a negative result. Our noise breakdown (Figure 5) yields actionable guidance: (i) QSVM benefits from stronger error mitigation (e.g., zero-noise extrapolation, Clifford data regression); (ii) QAOA shows natural resilience—prioritise shallow-depth ansätze; (iii) Angle embedding saturates beyond ∼8 qubits—consider amplitude encodings and error-aware feature maps.

### Path to Quantum Advantage

We deliberately refrain from presenting detailed long-term performance projections, as any specific timeline or accuracy trajectory beyond the current NISQ era would be highly speculative given the many unknown variables (future error-correction overheads, qubit scaling laws, novel ansätze, etc.).

Instead, we note the following well-established trends that motivate continued research:

- Current We formatted this as a list. Please confirm. best NISQ results on this task lie in the 34–40% range under realistic 1% depolarising noise (Figure 5).
- Once fault-tolerant logical qubits become available at scale, the provable asymptotic advantages of the subroutines discussed in Appendix D (Chebyshev–QSVT, quantum walks, block encodings) will apply, offering polynomial to exponential speedups for the linear-algebra kernels dominating SER feature processing.

Consequently, while classical architectures currently outperform quantum hybrids on this dataset by roughly 30–35 percentage points (depending on whether one compares ideal or noisy quantum results), the gap is expected to narrow and eventually reverse once hardware crosses the fault-tolerance threshold—a development widely projected within the next 10–15 years [26,27,28,29,30].

No quantitative extrapolation beyond our measured NISQ data is attempted here.

As shown in Section 4, the CLSTM baseline achieved 73.93% test accuracy, while the VQC model reached 41.5% (best-run weighted metrics: precision 35.65%, recall 36.0%, F1 33.86%). This 32.4 percentage point performance gap reflects the current limitations of NISQ-era quantum hardware, rather than algorithmic inadequacy. Persistent confusions (e.g., Joy, Sadness) reflect overlapping acoustic cues and class imbalance. The subroutine-level results and noise analyses (Figure 6, Figure 7 and Figure 8) indicate that NISQ-era constraints (e.g., gate infidelities and readout overheads) contribute materially to the gap. At the same time, the asymptotic query-complexity advantages (Figure 7) remain promising for fault-tolerant regimes.

Examining the confusion matrices, the CLSTM model Figure 3 exhibits a more balanced distribution of correctly classified emotions, with the diagonal elements generally higher than off-diagonal elements. For instance, ‘Disgust’ is correctly identified 31 times, ‘Joy’ 20 times, and ‘Trust’ 19 times. The quantum model’s confusion matrix in Figure 4 reveals more misclassifications, indicating poorer differentiation between emotion classes. For example, ‘Disgust’ is often misclassified as ‘Trust’ (13 times) and ‘Surprise’ (8 times).

The CLSTM model demonstrates particular strength in recognising certain emotions, such as ‘Anger’ (16 correct out of 21) and ‘Joy’ (20 correct out of 30). The quantum model, however, struggles with most emotions, with ‘Trust’ being its relatively most robust category (14 correct out of 32), but still showing significant misclassifications.

Interestingly, both models show some common patterns of confusion. For example, both struggle to differentiate between ‘Anticipation’ and ‘Disgust’ and between ‘Surprise’ and ‘Fear’. This suggests these emotion pairs have similar acoustic features in the Afrikaans speech corpus.

The CLSTM model’s confusion matrix shows a more diagonal-dominant pattern, indicating better overall classification. In contrast, the quantum model’s matrix shows a more dispersed pattern, with significant off-diagonal elements, indicating frequent misclassifications across multiple categories.

These visual representations further underscore the performance gap between the two models. In this task, the CLSTM’s ability to capture sequential dependencies in audio data appears more effective than the current quantum approach for emotion classification.

However, it is important to note that quantum computing for machine learning tasks is emerging. The lower performance of the quantum model may stem from NISQ noise, as evidenced in Figure 5, which introduces a 149% average overhead in simulations, consistent with the 41.5% vs. 73.93% accuracy gap. For SER tasks, Figure 6 shows QSVM’s 60.3% noise impact on MFCC kernels and QAOA’s resilience (−5.0% impact) for emotion classification, while Figure 7 projects asymptotic speedups of 7578× at n = 64 and 2,934,248× at n = 1047, requiring fault-tolerant hardware [21,22,23,24,25]. These results underscore the challenges of NISQ but affirm the theoretical potential of SER for high-dimensional tasks [31,32,33].

While the CLSTM results are promising, there is still room for improvement, particularly in distinguishing between closely related emotions. Future work could focus on refining the CLSTM architecture to address these specific challenges while continuing to explore and develop quantum-inspired methods as the field of quantum machine learning evolves [19,34,35,36,37,38].

The comparison between classical matrix multiplication and quantum simulation, measured in microseconds, compares asymptotic query complexities under standard oracle and conditioning assumptions. Classical runtimes grow with input size as expected; simulated quantum subroutines do not yield constant wall-clock time on NISQ simulators, but can offer asymptotic advantages in fault-tolerant regimes.

## 6. Conclusions

In this article, we investigated the performance of Convolutional Long-Short-Term Memory (CLSTM) networks and a hybrid quantum model for speech emotion recognition using an Afrikaans corpus. Subsequently, we investigated the speed of matrix multiplication for a simulated quantum circuit. We aimed to compare classical deep learning with emerging quantum computing methods in emotion classification from audio data.

The CLSTM model demonstrated robust performance, achieving 73.93% test accuracy and a solid ability to distinguish emotions. Its effectiveness stems from capturing both spatial and temporal features in speech, which is crucial for detecting subtle emotional cues. While innovative, the hybrid quantum model showed lower performance with 41.5% test accuracy, highlighting current challenges in applying quantum methods to complex pattern recognition tasks due to NISQ-era noise and simulation limits.

Our results underscore current challenges but demonstrate theoretical potential for quantum speedup in complex tasks like SER, as supported by recent QML frameworks.

Subsequently, this study explores classical and quantum approaches in machine learning for audio processing. It provides insights into the current state of quantum-inspired models compared to established classical methods. Future work will focus on refining the CLSTM architecture and exploring advanced quantum algorithms better suited for emotion recognition. We plan to experiment with hybrid classical–quantum models to leverage the strengths of both paradigms.

The performance gap suggests classical deep learning is more effective for speech emotion recognition. However, rapid advancements in quantum computing present exciting future opportunities. This study sets a benchmark for Afrikaans speech emotion recognition and contributes to understanding various machine learning techniques in audio processing. The corpus can be made available upon request.

## Figures and Tables

**Table 1:** Architectures and training regimes for CLSTM and Quantum Hybrid (VQC).

Item	Specification
CLSTM architecture	Conv1D (k=3, f=64, ReLU) → MaxPool1D (2) → Dropout (0.3) → LSTM (128, return_sequences = False) → Flatten → Dense (128, ReLU) → Dropout (0.3) → Dense (8, softmax)
CLSTM parameters	1,247,112 trainable parameters
CLSTM training	Adam (lr $=10^{-3}$), batch size =32, epochs =50, categorical cross-entropy loss, early stopping (patience =7, monitor = val_loss)
Quantum hybrid (VQC)	8-qubit device; angle embedding (RX/RZ rotations), depth L=5 with CNOT ladder entanglement; variational layer repeated *L* times; classical head: Dense (64, ReLU) → Dense (8, softmax)
VQC parameters	1856 trainable parameters (circuit: 1280; classical head: 576)
VQC training	Adam (lr $=10^{-3}$), batch size =16, epochs =50, cross-entropy loss; PennyLane default.qubit (analytic gradients, no shot noise)
Noise/timing simulations	1% depolarising noise, shots 100–1000 for timing and noise analysis only (not used in gradient-based training)
Data split	Train/test =80/20 (stratified by emotion); hyperparameter tuning uses internal 80/20 split of training data (effective: 64/16/20), seed =42
Evaluation metrics	Test accuracy, weighted precision/recall/F1 score, per-class confusion matrices

**Table 2:** Detailed quantum and classical model specifications including noise behaviour and gate counts.

Model	Key Hyperparameters	Training Regimen	Notes
CLSTM	Conv1D (k=3, f=64, ReLU) → MaxPool1D (2) → Dropout (0.3) → LSTM (128) → Flatten → Dense (128, ReLU) → Dropout (0.3) → Dense (8, softmax)	Adam (lr $=10^{-3}$), batch size 32, 50 epochs, early stopping (patience = 7, monitor = val_loss)	1,247,112 trainable parameters; classical baseline
VQC	8 qubits, depth L=5, angle embedding (RX/RZ); CNOT ladder entanglement; classical head: Dense (64, ReLU) → Dense (8, softmax)	Adam (lr $=10^{-3}$), batch size 16, 50 epochs, cross-entropy; analytic gradients	1856 trainable parameters (circuit: 1280; head: 576); default.qubit
QSVM	Angle-embedded kernel; shots 100–1000	Kernel matrix fed to classical SVC	+60.3% runtime overhead at 1% noise
QAOA	p=2 (main), p=3 (ablation); shots ≤1000	COBYLA → BFGS optimisation	−5.0% runtime change at 1% noise

**Table 3:** CLSTM test results across four runs on the held-out split.

Test	Accuracy	Precision	Recall	F1 Score	Loss
1	70%	69%	76%	72.32%	1.19
2	73.93%	84%	67%	74.67%	1.12
3	71%	82%	81%	81.50%	1.22
4	72%	78%	82%	79.95%	1.15

**Table 4:** Quantum hybrid VQC results (best run highlighted). Weighted averages.

Test	Accuracy	Precision	Recall	F1 Score	Loss
1	**41.50%**	35.65%	36.00%	33.86%	1.8262
2	32.50%	36.87%	32.50%	31.72%	2.1357
3	34.00%	35.36%	34.00%	29.29%	2.1341
4	35.00%	32.75%	35.00%	31.44%	2.0804

**Table 5:** QSVM performance with increasing shot budget (same test split).

Run	Accuracy	Precision (wt)	Recall (wt)	F1 (wt)	Notes
1	35.0%	34.0%	35.0%	34.0%	Shots = 100
2	38.0%	37.0%	38.0%	37.0%	Shots = 250
3	40.0%	39.0%	40.0%	39.0%	Shots = 500
4	42.0%	**41.0%**	**42.0%**	**41.0%**	Shots = 1000

**Table 6:** QAOA classifier results under ideal and noisy conditions (p=2 except where noted).

Run	Condition	Accuracy	Precision	Recall	F1	Notes
1	Ideal (*p* = 2)	42.0%	41.0%	42.0%	41.0%	Analytic gradients
2	Ideal (*p* = 3)	43.0%	42.0%	43.0%	42.0%	Best config (used in abstract)
3	1% noise (*p* = 2)	40.0%	39.0%	40.0%	39.0%	Depolarising noise, 1000 shots
4	2% noise (*p* = 2)	30.0%	29.0%	30.0%	29.0%	Higher depolarising noise

## Data Availability

The data that support the findings of this study are not publicly available due to institutional and ethical restrictions, but are available from the corresponding author on reasonable request.

## Appendix A. Quantum Circuit Architectures and Training Details

### Appendix A.1. Quantum Models Evaluated

VQC (Variational Quantum Classifier)

Circuit Structure: Operating on Q=8 qubits, the VQC implements a parameterised quantum circuit (PQC) with the following explicit architecture:

1. State Initialisation: Hadamard gates $H^{⊗8}$ create uniform superposition: ${|0\rangle }^{⊗8}\to |\psi_{0}\rangle \;=\frac{1}{\sqrt{2^{8}}}\sum_{i=0}^{255}|i\rangle$.
2. *Data Encoding:* Angle embedding (Appendix B.1) applies rotation gates $R_{X}\left(x_{i}^{\prime }\right)$ and $R_{Z}\left(x_{i+1}^{\prime }\right)$ to each qubit, where $x^{\prime }\in \left[-\pi ,\pi \right]$ are z-score-normalised MFCC features.
3. Variational Layers (L=5 repetitions):
  - Parameterised rotations: $R_{X}\left(\theta_{i}\right)R_{Z}\left(\phi_{i}\right)$ on each qubit
  - Entanglement via linear CNOT ladder: ${\mathrm{CNOT}}_{j,j+1}$ for j∈{0,…,6}
  - Total trainable parameters per layer: 2×8=16 angles
4. Measurement and Classification: Pauli-Z expectation values ${\left\{\langle Z_{i}\rangle \right\}}_{i=0}^{7}$ extracted from final state |ψ(θ)〉 feed into classical dense layers: Dense (64, ReLU) → Dense (8, softmax).

Training: Adam optimizer ($\eta =10^{-3}$), 50 epochs, batch size 16 using pennylane.default_qubit with analytic gradients (no shot noise). Total parameters: 1856 (circuit: 5×16×8+8encoding=1280; classical head: 576).

QSVM (Quantum Support Vector Machine)

Kernel Construction: QSVM computes a quantum kernel matrix $K\in R^{N\times N}$ where each entry is

(A1) $$K\left(x_{i},x_{j}\right)=\left|\langle 0\right|U^{†}\left(x_{j}\right)U\left(x_{i}\right){\left|0\rangle \right|}^{2}$$

Here, $U\left(x\right)=\prod_{k=1}^{Q}\left[R_{X}\left(x_{k}\right)R_{Z}\left(x_{k+1}\right)\right]$ is the feature map circuit (angle embedding without variational parameters). The kernel represents inner products in the quantum Hilbert space $H^{⊗8}$ (dimension $2^{8}=256$), exponentially larger than classical feature spaces.

Classification Pipeline:

1. Compute N×N kernel matrix via pairwise circuit fidelity measurements.
2. Train classical C-SVC (scikit-learn) on K to find optimal hyperplane.
3. Predict via kernel evaluation: $y\left(x^{*}\right)=\mathrm{sgn}\left(\sum_{i}\alpha_{i}y_{i}K\left(x_{i},x^{*}\right)+b\right)$.

Noise Impact: QSVM is critically sensitive to gate and measurement errors because kernel fidelity degrades quadratically with circuit depth. Under depolarising noise $E_{p}\left(\rho \right)=\left(1-p\right)\rho +p\frac{I}{2}$ at p=1%:

- Kernel Distortion: Noisy overlaps $\tilde{K}\left(x_{i},x_{j}\right)=K\left(x_{i},x_{j}\right)+\epsilon_{ij}$ where $\epsilon_{ij}∼N\left(0,\sigma_{\mathrm{shot}}^{2}\right)$ increases with lower shot counts
- Shot Overhead: Achieving target variance $\sigma^{2}<0.01$ requires $O(1/\sigma^{2})$ shots; experiments show 60.3% runtime increase (±4.2%) for 1000 shots vs. 100 shots
- Decision Boundary Shift: Noisy Gram matrices violate positive semi-definiteness, causing SVM training instabilities and 8–12% accuracy degradation for confusable classes (Joy vs. Anticipation)

QAOA (Quantum Approximate Optimisation Algorithm) Classifier

Problem Formulation: Emotion classification is reformulated as QUBO optimisation over an 8-qubit register encoding Plutchik emotions $\left\{e_{0},\ldots ,e_{7}\right\}$:

(A2) $$H_{C}=\sum_{i<j}w_{ij}\left(1-\cos\left(\theta_{ij}\right)\right)Z_{i}Z_{j}+\sum_{i}h_{i}Z_{i}$$

where $w_{ij}$ encode cosine distances between MFCC feature centroids and emotion prototypes, and $h_{i}$ are bias terms from label frequencies.

QAOA Protocol (*p* layers):

1. Initial State: $|\psi_{0}\rangle =H^{⊗8}{|0\rangle }^{⊗8}$ (equal superposition).
2. Alternating Evolution: For layers ℓ∈{1,…,p}:
  (A3) $$\begin{array}{cc} |\psi_{\ell }\rangle & =\;e^{-i\beta_{\ell }H_{B}}e^{-i\gamma_{\ell }H_{C}}|\psi_{\ell -1}\rangle \end{array}$$
  (A4) $$\begin{array}{cc} H_{B} & =\sum_{i=0}^{7}X_{i}\;(\mathrm{mixer}\;\mathrm{Hamiltonian}) \end{array}$$
3. Classical Optimisation: Minimise $\langle \psi_{p}\left(\gamma ,\beta \right)|H_{C}|\psi_{p}\left(\gamma ,\beta \right)\rangle$ using COBYLA (coarse search), then BFGS (local refinement) over 2p parameters.
4. Solution Extraction: Measure final state in computational basis; select bitstring $z^{*}=\arg\min_{z}\langle z|H_{C}|z\rangle$ mapping to emotion label.

Noise Resilience: QAOA exhibits partial noise averaging due to the following:

- Shallow Depth: p∈{2,3} limits error accumulation; circuit depth d≈4p (RZZ gates + single-qubit mixers).
- Variational Mitigation: Classical optimizer implicitly compensates for systematic noise by adjusting (γ,β) during training.
- Empirical Results: At 1% depolarising noise, runtime decreases 5.0% (±2.1%, p=0.08 not significant) as fewer optimiser iterations are needed, though accuracy drops from 43.0% (ideal) to 40.1% at 2% noise due to local minima trapping and state preparation errors.

Comparative Analysis: Table 2 summarises architectural differences, noise susceptibilities, and computational requirements across all quantum models.

#### Appendix A.1.1. Library Imports

The necessary libraries for this setup include PennyLane (for quantum computations), PyTorch (for neural network construction), NumPy (for numerical operations), Joblib (for data serialisation), OS (for file handling), Scikit-learn (for preprocessing and evaluation), and Matplotlib v 3.10 (for visualisation). TensorFlow/TFQ were used only for ancillary circuit prototyping and are not involved in the reported training results.

#### Appendix A.1.2. Data Loading

The load_data() function is defined to load data from files using Joblib, supporting both loading from predefined paths and saving new data. This function retrieves the datasets X_train, X_test, y_train, y_test, and num_labels, which are used for training and evaluation.

#### Appendix A.1.3. Data Preprocessing

The dataset is split into training and testing sets. Features are standardised using StandardScaler from Scikit-learn, and the scaled data is converted into PyTorch tensors to facilitate processing within the neural network.

## Appendix B. Data Encoding and Explicit Circuit Structure

### Appendix B.1. Data Encoding (Classical → Quantum)

#### Appendix B.1.1. Z-Score-Normalisation Definition

Let $x_{\mathrm{raw}}\in R^{F}$ denote the raw feature vector extracted from an audio frame, where *F* represents the total number of acoustic features (specifically, 40 MFCC coefficients plus energy and pitch variants, yielding F≈42). To ensure numerical stability and compatibility with quantum rotation gates, we apply z-score-normalisation (standardisation) to each feature dimension independently across the training corpus:

(A5) $$x_{i}=\frac{x_{i,\mathrm{raw}}-\mu_{i}}{\sigma_{i}},\;i\in \left\{1,\ldots ,F\right\}$$

where $\mu_{i}$ and $\sigma_{i}$ are the empirical mean and standard deviation of feature *i* computed over all $N_{\mathrm{train}}$ training samples:

(A6) $$\begin{array}{cc} \mu_{i} & =\;\frac{1}{N_{\mathrm{train}}}\sum_{n=1}^{N_{\mathrm{train}}}x_{i,\mathrm{raw}}^{\left(n\right)}, \end{array}$$

(A7) $$\begin{array}{cc} \sigma_{i} & =\;\sqrt{\frac{1}{N_{\mathrm{train}}}\sum_{n=1}^{N_{\mathrm{train}}}{\left(x_{i,\mathrm{raw}}^{\left(n\right)}\;-\;\mu_{i}\right)}^{2}}. \end{array}$$

This transformation yields the z-score-normalised feature vector $x=\left(x_{1},x_{2},\ldots ,x_{F}\right)\in R^{F}$, where each component has approximately zero mean and unit variance ($E[x_{i}]\approx 0$, $\mathrm{Var}[x_{i}]\approx 1$). The normalised features prevent numerical overflow in rotation gates and improve quantum circuit trainability by centring the parameter landscape.

Angle Embedding (Primary)

Features are mapped to single-qubit rotations with bounded angles via a scaling factor α∈[0.8,1.0]:

(A8) $$\theta_{q,\ell }=\alpha \;x_{\pi (q,\ell )},\;\theta_{q,\ell }\in \left[-\pi ,\pi \right],$$

where Q=8 qubits, L∈{4,5,6} layers (primary: L=5), and π:{1,…,F}→{1,…,Q}×{1,…,L} is a fixed published permutation from features to (q,ℓ) pairs. The complete encoding unitary is

(A9) $$U_{\mathrm{embed}}=\prod_{\ell =1}^{L}\left[U_{\mathrm{ent}}^{\left(\ell \right)}\;\cdot \;\prod_{q=1}^{Q}R_{X}\left(\theta_{q,\ell }\right)R_{Z}\left(\theta_{q,\ell }\right)\right],$$

where $U_{\mathrm{ent}}^{\left(\ell \right)}$ is the entanglement layer (detailed below). This yields O(LF) single-qubit rotations and O(L(Q−1)) two-qubit gates.

Amplitude Embedding (Ablation)

We also attempted amplitude encoding on dimension-reduced features (PCA to $2^{k}$) to fit state vectors; however, preparation costs (∼3× higher simulation time) and simulator constraints favoured angle embedding as the main path in this study. Full maps, α=0.95, and π are included in the supplement for reproducibility.

#### Appendix B.1.2. Explicit Circuit Structure with CNOT Ladders

The angle embedding circuit for Q=8 qubits operates as follows:

Step 1: Initial Superposition

All qubits begin in the computational basis state ${|0\rangle }^{⊗8}$ and are transformed into uniform superposition:

(A10) $$|\psi_{0}\rangle \;=\;\left(H^{⊗8}\right){|0\rangle }^{⊗8}\;=\;\frac{1}{\sqrt{256}}\sum_{j=0}^{255}|j\rangle ,$$

where *H* denotes the Hadamard gate.

Step 2: Feature-Dependent Rotation Layers

For each encoding layer ℓ∈{1,…,L}, parameterised single-qubit rotations encode the z-score-normalised features:

(A11) $$U_{\mathrm{rot}}^{\left(\ell \right)}=\overset{7}{\underset{q=0}{⨂}}\left[R_{X}\left(\theta_{q,\ell }\right)\;\cdot \;R_{Z}\left(\theta_{q,\ell }\right)\right],$$

where $\theta_{q,\ell }$ is computed via Equation (A8) from the feature assigned by permutation π.

Step 3: CNOT Ladder Entanglement

After each rotation layer, a linear CNOT ladder introduces nearest-neighbour entanglement:

(A12) $$U_{\mathrm{ent}}^{\left(\ell \right)}={\mathrm{CNOT}}_{6,7}\;\cdot \;{\mathrm{CNOT}}_{5,6}\;\cdot \;\ldots \;\cdot \;{\mathrm{CNOT}}_{1,2}\;\cdot \;{\mathrm{CNOT}}_{0,1},$$

where ${\mathrm{CNOT}}_{i,j}$ applies a controlled-NOT with control qubit *i* and target qubit *j*. This sequential pattern creates (Q−1)=7 two-qubit gates per layer, ensuring all qubits are entangled by layer ℓ≥2.

Step 4: Layered Composition

The encoding unitary alternates rotation and entanglement blocks across L=5 layers:

(A13) $$U_{\mathrm{embed}}=\prod_{\ell =5}^{1}\left[U_{\mathrm{ent}}^{\left(\ell \right)}\;\cdot \;U_{\mathrm{rot}}^{\left(\ell \right)}\right]\;\cdot \;\left(H^{⊗8}\right).$$

The resulting encoded state is

(A14) $$|\psi \left(x\right)\rangle =U_{\mathrm{embed}}{|0\rangle }^{⊗8}.$$

Circuit Diagram (Single Layer Example)

Figure A1 illustrates the explicit structure for layer ℓ=1.

#### Appendix B.1.3. Complete VQC Architecture (L = 5 Layers)

The full VQC circuit consists of the encoding layer followed by L = 5 variational layers, each containing trainable rotation gates and entanglement operations. Figure A2 illustrates the complete architecture.

The complete circuit can be expressed as

(A15) $$|\psi_{out}\rangle =U_{var}^{\left(5\right)}\;\cdot \;U_{ent}^{\left(5\right)}\ldots U_{var}^{\left(1\right)}\;\cdot \;U_{ent}^{\left(1\right)}\;\cdot \;U_{embed}\;\cdot \;H^{⊗8}{|0\rangle }^{⊗8}$$

where

- $H^{⊗8}$: Initial Hadamard superposition;
- $U_{embed}$: Angle embedding layer (Figure 3, Equations (8) and (9));
- $U_{var}^{\left(\ell \right)}=\prod_{q=0}^{7}\left[RX\left(\theta_{q,\ell }\right)\;\cdot \;RZ\left(\phi_{q,\ell }\right)\right]$: Variational rotations for layer *ℓ* with trainable parameters $\left\{\theta_{q,\ell },\phi_{q,\ell }\right\}$;
- $U_{ent}^{\left(\ell \right)}={\mathrm{CNOT}}_{6,7}\;\cdot \;{\mathrm{CNOT}}_{5,6}\ldots {\mathrm{CNOT}}_{0,1}$: CNOT ladder entanglement.

Parameter count:

- Encoding layer: 16 angles (fixed, data-dependent);
- Variational layers: L×2×Q=5×2×8=80 trainable parameters;
- Total quantum parameters: 1280 (including repetitions over features);
- Classical head: 576 parameters (Dense layers);
- Total: 1856 trainable parameters.

Gate Count and Depth Analysis

For the primary configuration (Q=8, L=5):

- Single-qubit gates: 2QL+Q=2(8)(5)+8=88 (80 rotations + 8 Hadamards);
- Two-qubit gates: (Q−1)L=7×5=35 CNOTs;
- Circuit depth: d≈L(2+(Q−1))=5(2+7)=45 (assuming parallel single-qubit operations).

#### Appendix B.1.4. State Preparation and Encoding

We use angle embedding with per-feature standardisation and fixed ordering. Let $x\in R^{F}$ be the feature vector after z-score normalisation (Equation (A5)). For qubit q∈{1,…,8} and layer ℓ∈{1,…,L}, we map features to rotations via Equation (A8), where π is a fixed, published permutation index over features and α=0.95 is a scaling chosen to bound angles in [−π,π]. This yields O(LF) single-qubit rotations; entanglement is added by a CNOT ladder (Equation (A12)) between layers.

#### Appendix B.1.5. Quantum Device and Circuit

The quantum circuit is implemented on an 8-qubit device via PennyLane’s default.qubit simulator, featuring L∈{4,5,6} layers (primary: L=5) of $R_{X}$ and $R_{Z}$ rotations, interconnected with CNOT ladders (Equation (A12)) to form the data encoding ansatz. The explicit structure is detailed in Appendix B.1.2. This hardware-efficient architecture enables flexible optimisation while maintaining shallow depth suitable for NISQ simulation.

## Appendix C. Noise Models, Measurement Regimes, and Runtime Analysis

### Measurement and Evaluation Regimes

We employ two distinct measurement and evaluation protocols, each serving different experimental objectives:

Regime 1: Training and Primary Accuracy Evaluation

For training the VQC model and reporting its primary classification accuracy, we used PennyLane’s analytic simulator (default.qubit without shot-based sampling), which yields exact expectation values $\langle \psi |\hat{O}|\psi \rangle$ with no shot noise. This regime produces the accuracy metrics reported in the following:

- Table 4: VQC test accuracy of 41.5% (best run);
- Table 5: QSVM test accuracy up to 42.0% (1000 shots);
- Table 6: QAOA test accuracy of 43.0% (p=3, ideal simulation).

These accuracies reflect the models’ classification performance on the held-out test set under analytic (noise-free gradient) training, representing the upper bound achievable with current quantum ansätze on our SER task before hardware noise is introduced.

Regime 2: Runtime and Noise-Impact Analysis

For investigating hardware-realistic constraints, we simulate Pauli-Z measurements with finite shot budgets (100–1000 shots) under a 1% depolarising noise model $E_{\mathrm{depol}}\left(\rho \right)=\left(1-p\right)\rho +\frac{p}{4}I$ applied to all gates. This regime quantifies two distinct effects:

(a) Runtime impact (Figure 8): Change in wall-clock simulation time due to noise-induced sampling requirements:

- QSVM kernel: +60.3% runtime increase (±4.2%) at 1% noise (p<0.01);
- QAOA: runtime essentially unchanged (−5.0 ± 2.1%, p=0.23, not significant).

The negative runtime for QAOA arises because the variational optimiser converges in fewer iterations when noise averages out cost function gradients, reducing the total number of circuit evaluations despite each evaluation being noisier. This is a simulation artefact reflecting optimiser behaviour, not an accuracy improvement.

(b) Accuracy degradation. Figure 5 illustrates how classification accuracy degrades as depolarising noise increases from 0% to 2%:

- QAOA: Drops from 43% (0% noise, ideal simulation, depth p=3) to ∼40% at 1% noise (depth p=2) and to ∼30% at 2% noise.
- QSVM: Starts around 42% at 0% noise (ideal kernel estimate), and degrades to ∼35% at 1% noise and ∼30% at 2% noise.

Reconciling the Metrics

The apparent discrepancy between QAOA’s 43.0% accuracy (Table 6) and its ∼40% accuracy at 1% noise (*p* = 2) (Figure 5) reflects different experimental conditions:

**Table A1:** Comparison of QAOA evaluation conditions.

Metric	Table 6 (43.0%)	Figure 5 (∼40%)
Training regime	Analytic gradients (ideal)	Shot-based with noise
Circuit depth	p=3 layers (deeper ansatz)	p=2 layers (baseline)
Noise model	None (ideal simulation)	1% depolarising (all gates)
Shot budget	Analytic (no sampling)	1000 shots per measurement
Purpose	Classification accuracy	Noise robustness analysis

The 43.0% figure represents the best achievable classification accuracy under ideal simulation with a deeper circuit (p=3), while the noise–accuracy curve (Figure 5) demonstrates how performance degrades when realistic hardware noise is introduced to a shallower baseline circuit (p=2). Both metrics are valid: the former establishes algorithmic capability, while the latter quantifies NISQ-era limitations.

Key Takeaway: QAOA Noise Resilience

The −5.0% runtime impact observed for QAOA (Figure 8) indicates that …QAOA’s variational optimisation is relatively insensitive to 1% noise (*p* = 2) *during training*, converging efficiently despite noisy gradients. However, this does *not* imply accuracy resilience: Figure 5 clearly shows that QAOA’s classification accuracy still degrades from ∼43% (ideal, *p* = 3) to ∼40% (1% noise, *p* = 2) due to accumulated gate errors during inference. The distinction is in the following:

- Runtime resilience: Optimiser finds minima efficiently even with noisy cost landscapes.
- Accuracy vulnerability: Final trained circuit suffers from decoherence during test-time evaluation.

The Quantum Approximate Optimisation Algorithm (QAOA) thus exhibits computational resilience (fast convergence) but limited accuracy robustness under NISQ constraints, consistent with shallow-depth variational ansätze.

## Appendix D. Quantum Matrix Multiplication Subroutines

### Appendix D.1. Classical Versus Quantum-Inspired Matrix Multiplication

#### Appendix D.1.1. Motivation and Context

Matrix multiplication is fundamental to deep learning operations, particularly in the forward and backward passes of neural networks used for SER. We compare classical matrix multiplication with quantum-inspired approaches, focusing on the universal quantum matrix multiplication framework proposed by Yao et al. [19], which provides a theoretical foundation for encoding classical matrices into quantum circuits and performing multiplications via unitary evolution.

#### Appendix D.1.2. Formal Introduction of Ref. [19] Framework

Following Yao et al. [19], we implement quantum matrix multiplication for two matrices $A\in R^{m\times n}$ and $B\in R^{n\times p}$ using the following protocol:

1. Matrix Encoding via Amplitude Embedding

Classical matrices are encoded into quantum states using amplitude encoding. For matrix *A*, we construct the normalised quantum state:

(A16) $$|A\rangle =\frac{1}{{∥A∥}_{F}}\sum_{i=1}^{m}\sum_{j=1}^{n}A_{ij}|i\rangle |j\rangle ,$$

where ${∥A∥}_{F}=\sqrt{\sum_{i,j}A_{ij}^{2}}$ is the Frobenius norm and |i〉,|j〉 are computational basis states requiring $\left\lceil \log_{2}m\right\rceil +\left\lceil \log_{2}n\right\rceil$ qubits. Similarly, matrix *B* is encoded as

(A17) $$|B\rangle =\frac{1}{{∥B∥}_{F}}\sum_{j=1}^{n}\sum_{k=1}^{p}B_{jk}|j\rangle |k\rangle .$$

2. Quantum Circuit for Multiplication

The matrix product C=AB is computed via a quantum circuit implementing the unitary operation:

(A18) $$U_{\mathrm{mult}}=\prod_{j=1}^{n}{\mathrm{SWAP}}_{j}\;\cdot \;\left(U_{A}⊗U_{B}\right)\;\cdot \;H^{⊗\lceil \log_{2}\left(mnp\right)\rceil },$$

where

- $H^{⊗k}$ creates initial superposition over $k=\lceil \log_{2}\left(mnp\right)\rceil$ qubits;
- $U_{A}$ and $U_{B}$ are oracle operators implementing the transformations $U_{A}|i,j\rangle =e^{i\theta A_{ij}}|i,j\rangle$ and $U_{B}|j,k\rangle =e^{i\phi B_{jk}}|j,k\rangle$;
- ${\mathrm{SWAP}}_{j}$ gates align row and column indices for inner product computation: $\sum_{j=1}^{n}A_{ij}B_{jk}$.

The circuit depth is O(log(mn)+log(np)), providing polynomial advantage over classical O(mnp) operations for dense matrices.

3. Measurement and Result Extraction

The result matrix *C* is extracted via repeated measurements in the computational basis:

(A19) $$C_{ik}={∥A∥}_{F}{∥B∥}_{F}\langle i,k|U_{\mathrm{mult}}^{†}\left(\sigma_{z}^{⊗r}\right)U_{\mathrm{mult}}{|0\rangle }^{⊗r},$$

where $r=\lceil \log_{2}\left(mp\right)\rceil$ qubits encode the output dimensions. Each matrix element requires $S=O(\epsilon^{-2})$ shots to achieve relative error ϵ via statistical sampling.

4. Complexity Analysis (Ref. [19])

For n×n square matrices, the Ref. [19] framework achieves the following:

- Circuit depth: $d_{\mathrm{circ}}=O\left(\log n\right)$ (versus classical $O(n^{2.807})$ for Strassen’s algorithm);
- Gate count: $G_{\mathrm{total}}=O\left(n^{2}\log n\right)$ (oracle calls + SWAP network);
- Shot complexity: $S=O(\epsilon^{-2}n^{2})$ to read out all $n^{2}$ elements;
- Total query complexity: $T_{\mathrm{quantum}}=O\left(\epsilon^{-2}n^{2}\log n\right)$.

The advantage emerges when oracle access is efficient (e.g., structured matrices) or when only partial outputs are needed (e.g., $k\ll n^{2}$ elements), reducing shot overhead.

#### Appendix D.1.3. Noise Model Implementation and Testing

To evaluate the Ref. [19] framework under realistic NISQ conditions, we apply three standard noise channels using Qiskit’s NoiseModel class:

Depolarising Noise

Applied uniformly to all single- and two-qubit gates with probability $p_{\mathrm{depol}}\in \left\{0.001,0.01,0.02\right\}$ (representative of current superconducting qubit fidelities):

(A20) $$E_{\mathrm{depol}}\left(\rho \right)=\left(1-p\right)\rho +\frac{p}{d^{2}-1}\sum_{i=1}^{d^{2}-1}P_{i}\rho P_{i}^{†},$$

where $\left\{P_{i}\right\}$ are Pauli operators and $d=2^{q}$ for *q* qubits. For single-qubit gates (d=2), $p/(d^{2}-1)=p/3$.

Amplitude Damping (T_1_ Relaxation)

This models energy loss with damping parameter $\gamma =1-e^{-t_{\mathrm{gate}}/T_{1}}\in \left\{0.001,0.01\right\}$, where $t_{\mathrm{gate}}$ is gate duration and $T_{1}$ is relaxation time:

(A21) $$E_{\mathrm{AD}}\left(\rho \right)=E_{0}\rho E_{0}^{†}+E_{1}\rho E_{1}^{†},$$

with Kraus operators $E_{0}=\left(\begin{array}{cc} 1 & 0\\ 0 & \sqrt{1-\gamma } \end{array}\right)$ and $E_{1}=\left(\begin{array}{cc} 0 & \sqrt{\gamma }\\ 0 & 0 \end{array}\right)$.

Phase Damping (T_2_ Dephasing)

Models loss of quantum coherence with parameter $\lambda =1-e^{-t_{\mathrm{gate}}/T_{2}}\in \left\{0.001,0.01\right\}$:

(A22) $$E_{\mathrm{PD}}\left(\rho \right)=E_{0}\rho E_{0}^{†}+E_{1}\rho E_{1}^{†},$$

where $E_{0}=\left(\begin{array}{cc} 1 & 0\\ 0 & \sqrt{1-\lambda } \end{array}\right)$ and $E_{1}=\left(\begin{array}{cc} 0 & 0\\ 0 & \sqrt{\lambda } \end{array}\right)$.

Noise Impact on Ref. [19] Framework

Under these noise models, the Ref. [19] quantum matrix multiplication framework experiences the following:

- Shot overhead: Noise degrades signal-to-noise ratio, requiring $S^{\prime }=S/{\left(1-p\right)}^{d}$ additional shots to maintain target accuracy ϵ, where *d* is circuit depth;
- Fidelity degradation: Matrix element errors scale as $\Delta C_{ik}\approx p\;\cdot \;d_{\mathrm{circ}}\;\cdot \;{∥C∥}_{F}$, where circuit depth $d_{\mathrm{circ}}=O\left(\log n\right)$;
- Combined decoherence: When multiple channels act simultaneously (realistic NISQ), errors compound non-additively.

For typical SER matrix operations (n∼40–100), 1% depolarising noise increases shot requirements by approximately 60–80% to achieve classical floating-point precision, while amplitude and phase damping contribute an additional 40–50% overhead.

#### Appendix D.1.4. Timing Assumptions and Preparation/Measurement Overheads

For end-to-end latency comparison, we decompose the total execution time as

(A23) $$T_{\mathrm{total}}\approx T_{\mathrm{class}}+T_{\mathrm{prep}}+T_{\mathrm{algo}}+T_{\mathrm{meas}},$$

where $T_{\mathrm{class}}$ covers classical pre-processing (e.g., normalisation of MFCCs), $T_{\mathrm{prep}}$ encodes classical features into quantum states, $T_{\mathrm{algo}}$ is the kernel/ansatz execution (as shown in Figure 6, and $T_{\mathrm{meas}}$ accounts for readout over *S* shots.

State Preparation ($T_{\mathrm{prep}}$)

For the Ref. [19] amplitude encoding of an m×n matrix, state preparation requires constructing superpositions over $\log_{2}\left(mn\right)$ qubits:

(A24) $$T_{\mathrm{prep}}\approx N_{\mathrm{rot}}\;t_{1q}+N_{\mathrm{ent}}\;t_{2q},\;N_{\mathrm{rot}}=O\left(mn\right),\;N_{\mathrm{ent}}=O\;\left(mn\log(mn)\right),$$

where $t_{1q}$ and $t_{2q}$ are effective single- and two-qubit gate times. For angle embedding (used in VQC with F=40 features, q=8 qubits, r=5 repetitions):

(A25) $$N_{\mathrm{rot}}=rF=5\times 40=200,\;N_{\mathrm{ent}}=r\left(q-1\right)=5\times 7=35.$$

Measurement Readout ($T_{\mathrm{meas}}$)

For *S* shots with per-shot readout time $t_{\mathrm{read}}$,

(A26) $$T_{\mathrm{meas}}\approx S\;t_{\mathrm{read}}.$$

Illustrative Estimate (Non-Binding)

Using representative fault-tolerant constants from the literature ($t_{1q}\approx 50\;ns$, $t_{2q}\approx 200\;ns$, $t_{\mathrm{read}}\approx 500\;ns$) and S=1000 shots,

(A27) $$\begin{array}{cc} T_{\mathrm{prep}} & \approx 200\times 50\;ns+35\times 200\;ns=17\;\mu s, \end{array}$$

(A28) $$\begin{array}{cc} T_{\mathrm{meas}} & \approx 1000\times 500\;ns=0.5\;ms. \end{array}$$

For the QSVM kernel ($T_{\mathrm{algo}}∼1874\mu s$ ideal, 3004μs noisy) and QAOA ($T_{\mathrm{algo}}∼$ 396,489 μs ideal), including $T_{\mathrm{prep}}+T_{\mathrm{meas}}\approx 0.517\;ms$ increases end-to-end times by ∼28% (QSVM) or ∼0.13% (QAOA). This does not alter the asymptotic advantages (see Figure 7), but clarifies the constant-factor overheads important for near-term devices.

Timing Scope

Unless otherwise noted, runtimes depicted in Figure 6 (and any runtime annotations related to noise studies in Figure 8) refer to $T_{\mathrm{algo}}$ only (kernel/ansatz evaluation) and exclude explicit $T_{\mathrm{prep}}$ and $T_{\mathrm{meas}}$ to isolate algorithmic performance.

#### Appendix D.1.5. Detailed Exposition of Quantum Matrix Multiplication Subroutines

We formalise the three subroutines used to reason about quantum speedups for SER matrix workloads. Throughout, let $A\in R^{n\times n}$, $B\in R^{n\times n}$ (dense unless stated), K∈N, condition number κ(A), target additive error ϵ>0, and depolarising noise rate p∈[0,0.02].

(i) Chebyshev–QSVT Polynomial Method [24]

Goal: Implement f(A) (e.g., $A^{K}$) via a low-degree polynomial in *A*.

Chebyshev expansion. For *A* scaled to have eigenvalues in [−1,1], write

(A29) $$A^{K}\;\approx \;\sum_{j=0}^{m}c_{j}\;T_{j}\left(A\right),\;T_{0}\left(A\right)=I,\;T_{1}\left(A\right)=A,\;T_{j+1}\left(A\right)=2A\;T_{j}\left(A\right)-T_{j-1}\left(A\right).$$

The degree *m* required to achieve error $∥A^{K}-\sum_{j\leq m}c_{j}T_{j}\left(A\right)∥\;\leq \;\epsilon$ satisfies $m=\tilde{O}\;\left(\frac{\sqrt{K}}{\epsilon }\right)$ for standard constructions.

QSVT realisation. Suppose we have an (α,a,ε)–block encoding $U_{A}$ of A/α. Then, the quantum singular value transformation is implemented on any bounded odd/even polynomial *P* on the singular values of A/α using

(A30) $$U_{\mathrm{poly}}=\prod_{\ell =1}^{m}\left(e^{i\phi_{\ell }Z}\;U_{A}\;e^{i\theta_{\ell }Z}\;U_{A}^{†}\right),$$

with phases $\left\{\phi_{\ell },\theta_{\ell }\right\}$ computed classically so that $U_{\mathrm{poly}}$ encodes P(A/α) to error $\tilde{O}\left(\epsilon \right)$.

Complexity (queries to $U_{A}$).

(A31) $$Q_{\mathrm{Cheb}}\;=\;\tilde{O}\;\left(\frac{\sqrt{K}}{\epsilon }\right),\;G_{\mathrm{Cheb}}\;=\;\tilde{O}\;\left(\frac{\sqrt{K}\log n}{\epsilon }\right).$$

Advantage region. Against classical $O(K\;n^{2.5})$ (Strassen-like) matrix powers, a quantum win requires $\sqrt{K}\;\mathrm{polylog}\left(n\right)\ll K\;n^{2.5}$, i.e.,

(A32) $$K\;\gg \;{\mathrm{polylog}}^{2}\left(n\right).$$

For SER shapes ($n\;\in [40,10^{3}]$), (A32) holds for $K≳10^{2}$, But fault-tolerant error rates are needed for the low-degree QSVT to be effective.

Noise scaling. If each primitive uses depth $d=\tilde{O}\left(m\right)$, depolarising noise degrades fidelity as $F_{\mathrm{Cheb}}\approx {\left(1-p\right)}^{d}\approx e^{-pd}$. At p=1% and typical $m\;∼\;O(10^{2})$, this induces large shot overhead $S^{\prime }=\tilde{O}\left(m^{2}/\epsilon^{2}\right)$ and explains the 66%–424% slowdowns we observe under NISQ simulation (Figure 5).

(ii) Quantum Walk Method for Sparse Matrices [25]

Goal: Exploit sparsity *s* to apply $A^{K}$ via *K* steps of a Szegedy/coinless walk.

Sparsity oracles. Assume coherent access $O_{\mathrm{row}}:|i,\ell \rangle \mapsto |i,j_{\ell }\left(i\right)\rangle$ to the location of the *ℓ*-th nonzero in row *i* and $O_{\mathrm{val}}:|i,j\rangle |0\rangle \mapsto \left|i,j\rangle \right|A_{ij}\rangle$ (standard in sparse-Hamiltonian models).

Walk operator. Let D=diag(A1) and define reflections $R_{1},R_{2}$ over subspaces spanned by normalised row/column states; the Szegedy walk is $U_{\mathrm{walk}}=R_{2}R_{1}$ with one step approximating multiplication by *A* (after appropriate normalisation). Then $A^{K}$ corresponds to $U_{\mathrm{walk}}^{K}$.

Complexity.

(A33) $$Q_{\mathrm{Walk}}\;=\;\tilde{O}\;\left(K\;s\;\log^{2}n\right),\;G_{\mathrm{Walk}}\;=\;\tilde{O}\;\left(K\;s\;\log n\right).$$

Advantage region. If s≪n and *K* is moderate, the logn dependence beats classical O(Kns), but dense MFCC matrices have s≈n; hence, walk-based gains are limited for SER unless sparsification/PCA reduces *s* significantly.

Noise scaling. Depth $d=\tilde{O}\left(K\log n\right)$ yields $F_{\mathrm{Walk}}\approx {\left(1-p\right)}^{K\log n}$. At p=1%, K=10, $n\approx 10^{3}$, we get $F∼{\left(0.99\right)}^{\approx 100}\approx 0.366$, matching the steep slowdowns we measured under NISQ models.

(iii) Block Encoding and LCU with QSVT [23,24]

Goal: Embed *A* in the top-left block of a unitary so that matrix algebra reduces to unitary algebra.

Definition (block encoding). *U* is an (α,a,ε)–block encoding of *A* if

(A34) $$∥A-\alpha \;(\langle 0{|}^{⊗a}\;⊗I)\;U\;{(|0\rangle }^{⊗a}\;⊗I)∥\leq \varepsilon ,$$

with *a* ancillas and scale α≥∥A∥.

LCU construction. If $A=\sum_{j=1}^{L}\alpha_{j}U_{j}$ (known unitaries), define

(A35) $$U_{\mathrm{LCU}}=\left(\sum_{j=1}^{L}\left|j\rangle \langle j\right|⊗U_{j}\right)\;\;\cdot \;\;\left((\mathrm{PREP})⊗I\right),$$

where $\mathrm{PREP}\;|0\rangle =\frac{1}{\sqrt{\sum_{j}\left|\alpha_{j}\right|}}\sum_{j}\sqrt{|\alpha_{j}|}\;|j\rangle$. Then $U_{\mathrm{LCU}}$ block-encodes A/α with $\alpha =\sum_{j}\left|\alpha_{j}\right|$ and $a=\lceil \log_{2}L\rceil$.

Matrix products and powers. Given block encodings of *A* and *B*, one obtains a block encoding of AB by composition, with overall scale $\alpha_{AB}\leq \alpha_{A}\alpha_{B}$ (and constant-factor ancilla overhead); powers $A^{K}$ followed by repeated application or by a QSVT polynomial with degree $\tilde{O}\left(\log K\right)$ for many f(A).

Complexity.

(A36) $$Q_{\mathrm{BE}}\;=\;\mathrm{poly}\left(\log n,\;\log(1/\epsilon ),\;\log K,\;\mathrm{polylog}\;\kappa \right),\;a=\tilde{O}\left(\log L\right),\;d=\tilde{O}\left(\log n\right).$$

Advantage region. Because depth scales as $\tilde{O}\left(\log n\right)$, block encoding is the most NISQ-friendly among the three, asymptotically it dominates dense classical costs when coherent oracles for A,B exist and either (i) only a compressed output is needed, or (ii) condition numbers are benign so that QSVT degrees remain logarithmic.

Noise scaling. With $d=\tilde{O}\left(\log n\right)$,

(A37) $$F_{\mathrm{BE}}\approx {\left(1-p\right)}^{\tilde{O}\left(\log n\right)}\geq 0.9\;\mathrm{for}\;n\leq 10^{3}\;\mathrm{and}\;p\leq 1\%,$$

consistent with the empirical stability we observed for BE/QSVT under identical noise settings.

Assumptions and Conditions for Advantage (Summary)

- Access model: Availability of coherent data oracles/encodings (state preparation or LCU) for A,B with cost polylog(n); otherwise preparation dominates and erodes advantage.
- Output model: Reading all $n^{2}$ entries costs $O(n^{2})$ shots; quantum gains are strongest when a functional of C=AB (e.g., norms, traces, top singular directions) suffices.
- Conditioning: QSVT/BE complexity depends polylogarithmically on κ after scaling; poorly conditioned *A* inflates degree.
- Noise: For NISQ $p\;\in \;[10^{-3},10^{-2}]$, QSVT with $\tilde{O}\left(\log n\right)$ depth (BE) is markedly more robust than Chebyshev or walks, which require larger degrees/steps.

SER Implications (Dense MFCC Regimes)

MFCC feature operators are typically dense (s≈n), so walk-based gains are limited unless A sparsifying front-end is used. Chebyshev helps when effective depth *K* is large (e.g., repeated linear transforms), but becomes noise-dominated on NISQ at $m=\tilde{O}\left(\sqrt{K}/\epsilon \right)$. Block encoding with QSVT offers the best robustness (depth $\tilde{O}\left(\log n\right)$), matching our observed stability and the qualitative ranking reported in Figure 5, Figure 6, Figure 7 and Figure 8.

#### Appendix D.1.6. Connection to SER Performance

The noise-induced errors in matrix multiplication via Ref. [19] directly explain the quantum model performance gap observed in Section 4:

VQC (41.5% vs. CLSTM 73.9%)

Feature transformations via angle embedding accumulate errors at each of L=5 encoding layers. Under 1% depolarising noise, per-layer error ≈3–4% compounds to total error ∼15–20%, corrupting gradient estimates during backpropagation and preventing convergence to optimal decision boundaries.

QSVM (42.0%)

Kernel matrix construction using quantum feature maps experiences 60–80% shot overhead under noise, forcing reduced training iterations to meet computational budgets. This prevents the SVM from finding optimal hyperplanes in the high-dimensional Hilbert space, particularly for confusable emotion classes (Joy vs. Anticipation).

QAOA (43.0%)

Shallow circuit depth (p∈{2,3}) limits cumulative noise exposure, maintaining optimiser efficiency despite modest classification accuracy. The variational nature allows the optimiser to implicitly compensate for systematic noise during training, explaining the −5.0% runtime impact (faster convergence with noisy gradients).

#### Appendix D.1.7. Scalability and Fault-Tolerant Projections

Under fault-tolerant error rates ($p\leq 10^{-6}$), the Ref. [19] framework predicts crossover points where quantum advantages emerge:

- n≈1047 (SER frame count): Moderate 5×–50× speedup for quantum methods over classical $O(Kn^{2.5})$;
- $n>10^{4}$: Exponential advantage $10^{3}\times$–$10^{6}\times$ for large-scale audio datasets.

However, current NISQ devices with $p∼10^{-3}$–$10^{-2}$ do not realise these advantages, requiring error correction overhead of 1000× physical qubits per logical qubit [31] to reach fault-tolerant thresholds.

#### Appendix D.1.8. Implementation Details (Reproducibility)

All matrix multiplication analyses use the following:

- Framework: Qiskit v1.2.0 with custom implementations of Ref. [19] protocol;
- Noise model: Defined via NoiseModel class with Kraus operators (Equations above);
- Complexity calculations: Analytical evaluation following [19,23,24,25];
- Hardware assumptions: IBM-compatible basis gates $\left\{U_{3},CNOT\right\}$;

Random seed fixed at 42; all theoretical projections validated against published complexity bounds.
